# Supplementary material for: Leveraging interindividual variability in threat conditioning of inbred mice to model trait anxiety
Source: PLoS Biol. 2024 May 28;22(5):e3002642. doi: 10.1371/journal.pbio.3002642 (PMC11161093; doi:10.1371/journal.pbio.3002642)
Supplement: Attachment — Submitted filename: PBIOLOGY-D-23-02481R1_Point-by-point letter.pdf [file pbio.3002642.s021.pdf]

Reviewer#1:

In this paper by Kovlyagina and colleagues, the authors aim to determine trait-like anxiety phenotypes in female and male C57BL6/J mice based on differences in their freezing response during memory retrieval in an auditory aversive conditioning (AAC) task. The authors distinguish animals into phasic and sustained responders based on their freezing profile during memory retrieval and find most individual differences in the sustained freezing response phase. The authors then correlate the individual sustained freezing responses with other measures of anxiety-like behavioral phenotypes, such as avoidance in the EPM, OF, or NOR tasks. Further transcriptomic and electrophysiological experiments reveal interesting gene expression and neurophysiological differences between sustained and phasic responder mice that are relevant for the study of psychiatric disorders. Overall, I think this is an interesting study that proposes a new approach to stratify (inbred) mice based on inferences on their trait anxiety derived from freezing responses during fear conditioning. The following points should be addressed:

Reply: We thank the reviewer for acknowledging the strengths of our study and appreciating the level of detail of our analyses.

To understand if the "trait" anxiety phenotype is a true "anxiety"-like response or a difference in cognitive function that correlates with measures of avoidance, two questions should be addressed:

1. Do sustained vs phasic responders show differences in their response to the shock during memory encoding? If so, are there differences in e.g. pain perception between these two groups of mice that affect their sensitivity to the shock and therefore the strength of the encoded memory?

Reply: We have not specifically examined animals for pain reactivity, since multiple studies involving mice, rats and cats have shown unambiguously that differences in pain reactivity do not correlate with freezing behaviour during MR (Lehner et al. 2010; Sartori et al. 2011; Werka 1980). Lehner et al., specifically addressed this question using rats and found no correlations between pain sensitivity measured by two different tests (flinch-jump, tail flick) and freezing behaviour induced by novelty or conditioned freezing. Moreover, authors found no correlation between results of “flinch-jump” and “tail flick” tests indicating “that the behavioural pain expression is not a reliable indication of pain perception”(Lehner et al. 2010). Additionally, it has been shown that high and normal anxiety selective breeding mouse lines do not differ from each other in “flinch-jump” test either (Sartori et al. 2011).

We observed a strong freezing response during the initial bin of CS presentation (bins 13) without significant differences between sexes and sustained and phasic freezers across all investigated batches (Main Text Figures 2d, e, Extended Data Figure 1d, 2d, 2e, 3f, 3g, 5d, 5e). Based on these observations we concluded that all animals similarly learned the association between the tone and foot shock (similar strengths of memory acquisition). In our manuscript, we operate within a framework that the phasic component of MR reflects memory acquisition, supported by evidence from various studies using associative learning paradigms. In contrast, we argue that differences in freezing behaviour during the sustained

phase do not arise from variations in learning but rather from **individual differences in the perception of imminence of threat**., e.g. interpretation of the CS predictive value, defined by inborn anxiety trait.

2. Do sustained vs phasic responders differ in other memory-related task that are not based on fear conditioning?

Reply: We performed the Novel Object Recognition (NOR) in the initial phases of establishing our behavioural paradigm (Reviewer Figure 1). While we noted a strong correlation between sustained freezing (bins 18-24) and both frequency of approach and total time spent with objects on both testing days (Reviewer Figure 1a-f), no significant correlation was detected for the discrimination index (Reviewer Figure 1g). Furthermore, there were no significant differences between naïve animals, sustained and phasic responders in the discrimination index (Reviewer Figure 1h) This observation suggested that sustained responders were more reluctant to approach the objects. Consequently, we replaced NOR with the Novel Object Exploration (NOE) test in the following experiments. Additionally, given that emotional and non-emotional memories are governed by distinct brain circuitries, our primary objective was to establish a comprehensive framework for assessing animals' anxiety trait. As a result, our focus was directed towards emotional learning and emotionally-motivated approach-avoidance tasks.

Moreover, how do the authors exclude the possibility that the gene expression and electrophysiological profiles identified in their study are not a result of the differences in fear conditioning, rather than a reflection of trait anxiety differences, considering that tissue was obtained after AAC and behavioral testing?

Reply: It is inherently not possible to disentangle whether transcriptomic differences in phasic versus sustained responders originate from differences in trait anxiety or from differences in cellular events induced by the AAC and the memory retrieval, as it is not possible to collect tissue samples before and after AAC. Most likely, the observed differences are a reflection of both factors. Nevertheless, we are arguing that differences in aversive conditioning are a direct consequence of differences in trait anxiety. Moreover, we estimate that the samples collected for the second RNA-seq, 30 minutes after MR2 (28 days post-conditioning), do not provide sufficient time for transcription completion, except for immediate early genes that do not require *de novo* protein synthesis (Fowler, Sen, and Roy 2011; Phillips et al. 2023). Therefore, the **RNA-seq results post-MR2 are more likely to mirror the baseline differences between phasic and sustained responders**. It is interesting to note that the similarity of our dataset with the human PTSD transcriptome is highest at MR2. This may support the claim that the transcriptome at MR2 reflects a state after long-term "incubation" of a strong aversive conditioning, similarly to what occurs in the human pathology of PTSD.

Furthermore, electrophysiological recordings were performed 1 month after the last MR and 2 months after conditioning. Neurons for patching were selected randomly. Neurons for patching were selected randomly. Consequently, we believe these recordings offer insights into baseline differences in excitability between phasic and sustained responders. **In**

essence, acquiring baseline brain tissue for RNA-seq or *ex vivo* recordings without the confounding impact of AAC proves unfeasible, given the necessity to first phenotype the animals. Baseline differences can be only understood using non-invasive or *in-vivo* experimental techniques (experiments in progress).

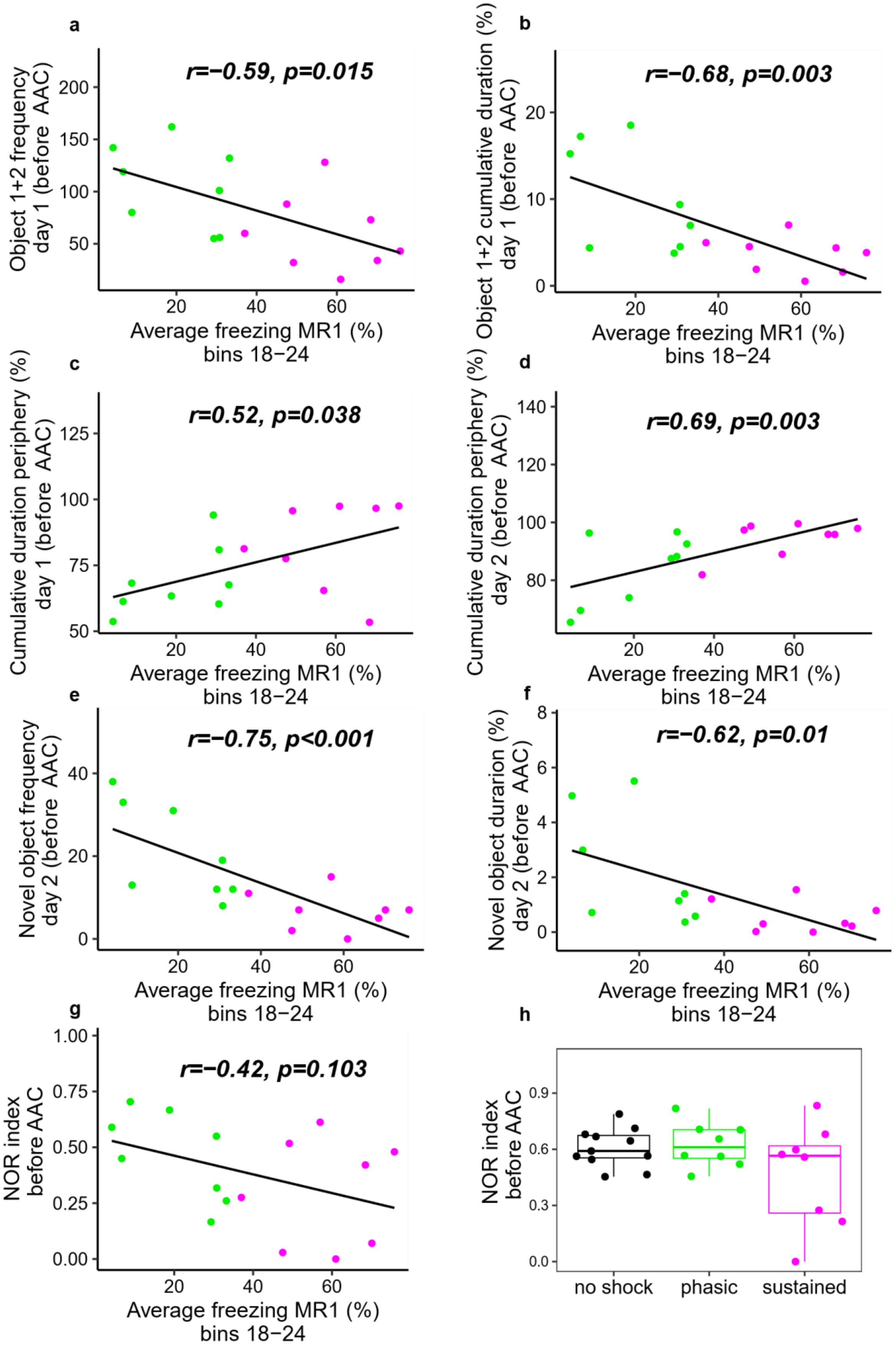

Reviewer Figure 1. Novel object recognition (NOR) test was performed 1 week prior to AAC to assess baseline differences in memory of phasic and sustained responders. **a-f**, Approach-avoidance

behavior during NOR correlated with sustained freezing during MR1. **g**, NOR index did not correlate with sustained freezing during MR1. **h**, No significant group differences were detected in NOR index

Reviewer #2:

Kovlyagina and colleagues aim to address in this manuscript the issue of inter-individual variability in behavioral stress responses. They argue that in contrast to the common practice of testing for group mean responses, for a high translational value of the studies to understand human psychopathologies individual differences and variabilities in the responses need to be considered. I fully agree with the authors that this is a highly relevant question to study. Additional issues addressed by the authors are the importance to include both sexes in these studies, and to provide the possibility for longitudinal observations over time. Based on these research aims the authors adopt a fear conditioning protocol with unpredictable CS-US timing and prolonged CS exposure during memory retrieval. They subsequently categorize the freezing response of the animals across the prolonged CS exposure and split the animals in two subgroups that show either sustained of phasic freezing responses. These groups of mice are then compared for their transcriptomic response in different brain regions as well as electrophysiological properties in the lateral amygdala. I have a number of concerns and comments, which I believe are critical for the proposed research question and which should be addressed by the authors:

Reply: We are grateful to the reviewer for highlighting the important aims of our study and acknowledging their relevance. We provide a detailed response to the reviewer's concerns below.

a. I believe it is not correct of the authors to treat fear and anxiety as interchangeable phenomenon. Indeed, while there is certainly an overlap of both behaviors in terms of underlying neurocircuitry, fear and anxiety are still fundamentally different behaviors driven by distinct neuronal circuits and mechanisms. Both can be correlated but are certainly not under all circumstances. Also, within the framework of the predatory imminence theory the responses related to fear and anxiety (and panic) are quite temporally distinct. This issue needs to be better addressed in the manuscript.

Reply: We completely agree with the reviewer regarding the nuanced distinction between the terms "fear" and "anxiety," as well as with the notion that they should not be used interchangeably. We paid great attention to it while writing the manuscript. As a matter of fact, we have not used the word "fear" once in results and discussion. In the introduction, our reference to high- and low-fear animals was merely a reflection of the terminology used by the authors in the original publications we cited. We would like to emphasize that we referred to the behaviour we measured during MRs as a freezing response. The only conclusion we made was that the sustained component of the MR session reflected anxiety (approach-avoidance) behaviour (not emotion, i.e. fear) based on multiple significant correlations with classical anxiety tasks performance. In the scope of this manuscript, we were not dealing with the concept of fear.

b. A major drawback of the authors' approach is in my view the subdivision of the behavioral response in two subgroups: phasic and sustained responders. Instead of truly studying individual variability, the authors thereby merely group the behavioral response in two rather artificial sub-classes. There are multiple tools available to classify behavior of animals not only on a single behavioral readout (here freezing), but capture the high complexity of the behavioral repertoire of the animals over time, e.g. using markerless pose estimation combined with supervised as well as unsupervised behavioral analyses. Even when looking exclusively at the absence or presence of freezing behavior, the data clearly indicate that animals within the groups (phasic or sustained freezing) largely differ in the time course of the behavioral response and represent a continuum rather than two distinct groups. I therefore believe it will be imperative for the authors to implement a more in-depth behavioral phenotyping and utilize the true individual variability between the behavioral strategies of the animals as basis for their further analyses. With the current approach I don't see how the main aim of the study can be met.

Reply: We agree with the reviewer that the behavioural response is a continuum and not a categorical outcome. For this reason, we already extensively focused on correlational analysis.

However, individual based analysis also requires larger samples sizes to account for the additional random variability in continuous measures. We are limited in the number of animals we can include in the experiments mainly due to ethical but also practical reasons. Group level analysis can therefore help to boost statistical power.

We would also like to point out that **freezing is not a single readout but a class of measures including multiple time points**, freezing across different stages of CS presentation, as well as estimated parameters of the fitted freezing curves (intercept and decay rate). We did not randomly select the clustering parameters. In fact, we initially performed backward and forward greedy feature selection analyses with MR1 freezing parameters in three independent male and female cohorts as implemented in the clustvarsel R package to elucidate the most predictive variables. Average freezing bins 18-24 was identified as a predictive feature in all selection steps, and decay rate and intercept of the fitted freezing curve were selected in three out of the 4 iterations. Additional parameters were selected in no more than two models. Therefore, we ultimately performed clustering using average freezing bins 18-24, decay rate and intercept of the fitted freezing curve. We have included these details in the revised manuscript (lines 706-711).

While the reviewer correctly points out that there are numerous analysis strategies for integrating multiple behavioral measures, we would like to stress the trade-off between too many features leading to increased model complexity and generalizability of results.

Moreover, we did not aim at building a predictive model for individual behavioural readouts (e.g., freezing) which would likely lead to overfitting considering our sample sizes that, though larger than most behavioural studies, are still limited. Our central goal was to

operationalize trait anxiety using an interpretable behavioural marker that generalizes reliably in both sexes across multiple batches.

Importantly, the advantage of our pipeline is that it can be easily extended by additional modules such as continuous observation of animals in home cages. In fact, in a follow-up project we are performing such experiments to characterize our elucidated phenotypes longitudinally across different social interaction contexts. Here, tools such as markerless pose estimation could indeed be essential to identify novel behavioural features relating to trait anxiety. However, we believe that starting out with an even more complex observational system of animal behaviour which as pointed out by the reviewer would also necessitate the use of supervised (thus labelling and classification into groups) or unsupervised (clustering) methods, would produce results devoid of a theoretical framework.

c. I see a number of conceptional issues with the data and conclusions from the experiments depicted in Figure 2. With the aim of the study to look at individual differences and variability, the authors here fall back to compare group means of average freezing in 2 retrieval sessions and readouts of established anxiety tests like EPM or OFT. While the two stratified groups of phasic and sustained freezers differ in the anxiety readouts, the overlap of the data is huge and all focus on individual variability is lost. Correlation analyses are in my view essential here, but not across conditions (as done for OFT in Figure 2g and h) but within a condition. Even for the OFT data the correlations are relatively weak and would not allow a prediction of anxiety or freezing behavior for an individual animal.

Reply: As outlined above, while we provide extensive correlational analyses, we also additionally performed group level analysis as a mean of increasing statistical power. The observed overlap between the groups is indeed due to freezing behaviour being a continuum. According to the reviewer’s suggestion, we have calculated phenotype-specific correlations. We observed that the direction and magnitude of the associations are highly comparable for both sustained and phasic responders (Reviewer Figure 2). Therefore, within group correlation analyses would not change the conclusion of our manuscript, but it reduces statistical power.

As outlined above, the aim of our study was not to provide a predictive model for individual measures of individual animals. We take advantage of the inter-individual variability to assign animals to consistent endophenotypes according to their anxiety trait for subsequent investigation of molecular mechanisms underlying these differences. Due to the stochasticity in complex animal behaviours, variability within identified animal classes is still naturally expected, which would make exact prediction of an animal’s behaviour in an individual measure nearly impossible (Honegger and de Bivort 2018).

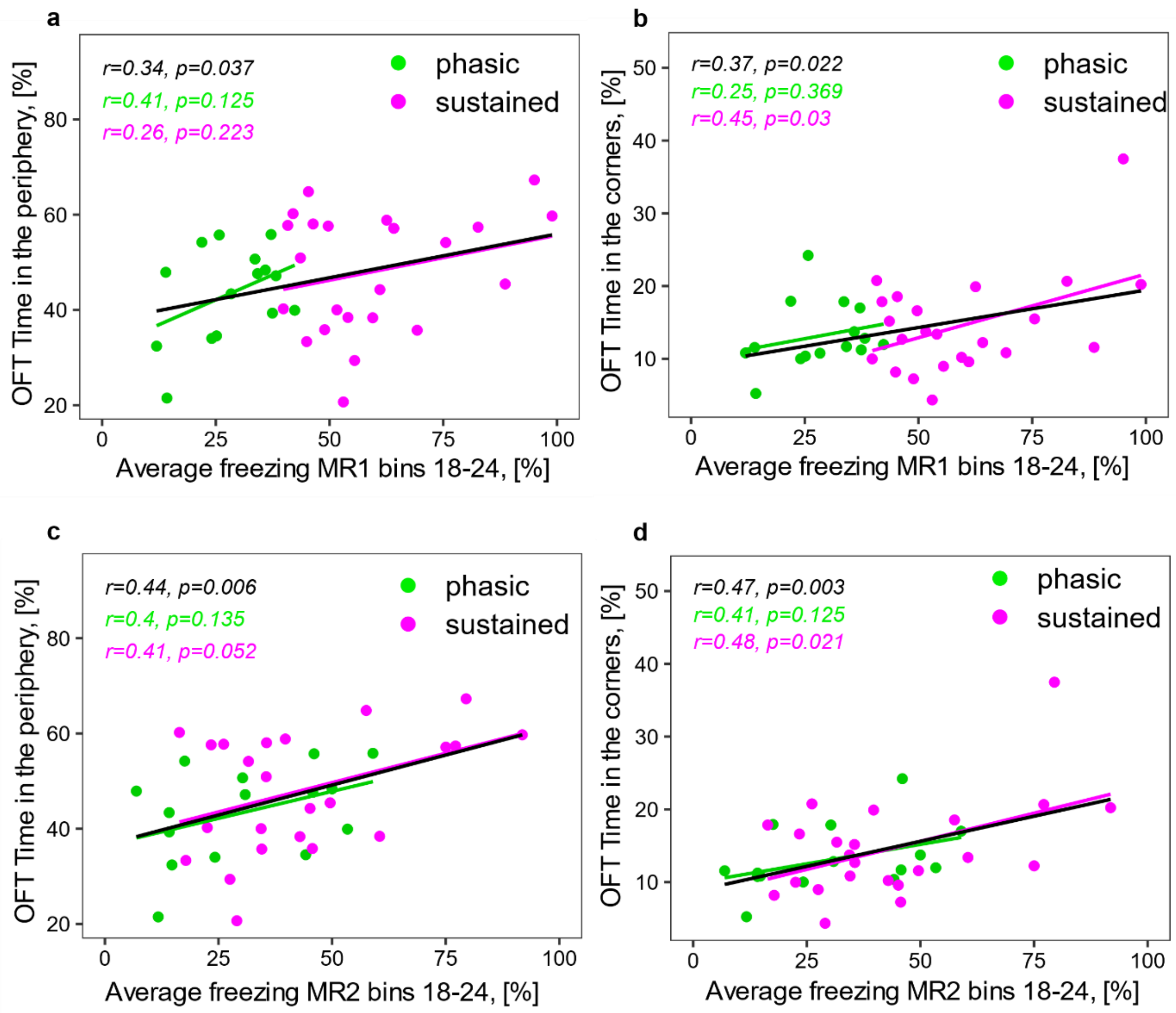

Reviewer Figure 2 Sustained freezing during MR1 and MR2 was significantly correlated with approach-avoidance behavior in the open field test (black trend lines) as indicated by the total time spent in the periphery **(a, c)** and in the corners **(b, d)**. Subgroup analysis of phasic (green line) and sustained responders (magenta line) indicated that the direction and magnitude of the correlation was similar within endophenotypes as in the overall population. r: Pearson correlation coefficient.

d. The advantage of using stratification of freezing behavior in a fear conditioning paradigm over classical tests is not apparent to me. The use of the EPM and a stratification of animals and high anxiety (avoiders of open arms) and low anxiety (explorers of the open arm) would have – according to the authors – resulted in very similar subgroups compared to the now selected phasic and sustained freezers. The EPM tests is a lot simpler compared to the fear conditioning paradigm, the readouts are easier to interpret, and the test is a lot less stressful compared to the fear conditioning paradigm, where a foot shock stressor is needed. The only argument in favor of using the paradigm proposed by the authors would be the high correlation of freezing behavior between different MR sessions. However, I am certain that similar correlations would be observed with subsequent tests of OFT or EPM. In addition, these tests can also be modified (different texture, smell, surrounding, etc) to increase novelty and under these circumstances can be repeatedly utilized in the same individuals without problems. As a consequence, with the currently presented data I am not

convinced of any additional benefit of using the fear conditioning and prolonged CS exposure paradigm. This might change if the authors adopted a deeper phenotyping approach (see my comment (b)), but for the detection of freezing behavior alone the methodological or conceptional advance of the applied approach is not evident.

Reply: We strongly believe that no single test can provide comprehensive information about an animals' trait. Traits, defined as consistent behavioral patterns within an individual across various contexts, **necessitate evaluation through diverse tasks for effective classification**. While conditioning tests offer full control over stimulus presentation that can be repeated a few times, setting them apart from unconditioned tasks (OFT, LDT, NOE), we think **the true strength of our approach lies in the combination of both conditioned and unconditioned tasks**. This approach allows robust classification of animals according to their anxiety trait for further investigation.

From the translational point of view, addressing different behavioral domains directly reflects approaches applied in the diagnosis of neuropsychiatric disorders, as given in DSM-5 (Diagnostic and Statistical Manual of Mental Disorders), where sub-groups of diagnostic criteria are formed. To be diagnosed a patient must check any few criteria from each group, not necessarily all criteria. Similarly, our method provides a nuanced evaluation that does not demand adherence to all conditions.

It was also important to provide a tool that is easy-to-adopt. In our work, we were aiming for the **necessary minimum of testing** to classify animals according to their anxiety trait while not compromising on the replicability of results. This pipeline can be easily supplemented with long-term observation cages, or certain tests can be analysed with software such as DeepLabCut or MotionSeq if research questions demand a higher resolution of behavioural data.

Moreover, we think this observation might be valuable not only for the stress-research community, but for anyone who is using aversive conditioning paradigms since some critical results might be lost through averaging over the entire cohort of conditioned animals as opposed to trying to identify distinct subgroups. Additionally, we tried to implement repeated testing for non-conditioned tasks such as OFT and NOR. We observed dramatic and significant reduction of activity overall in all groups of animals (including no shock control group), despite context modification and new sets of objects for NOR (Reviewer Figure 3).

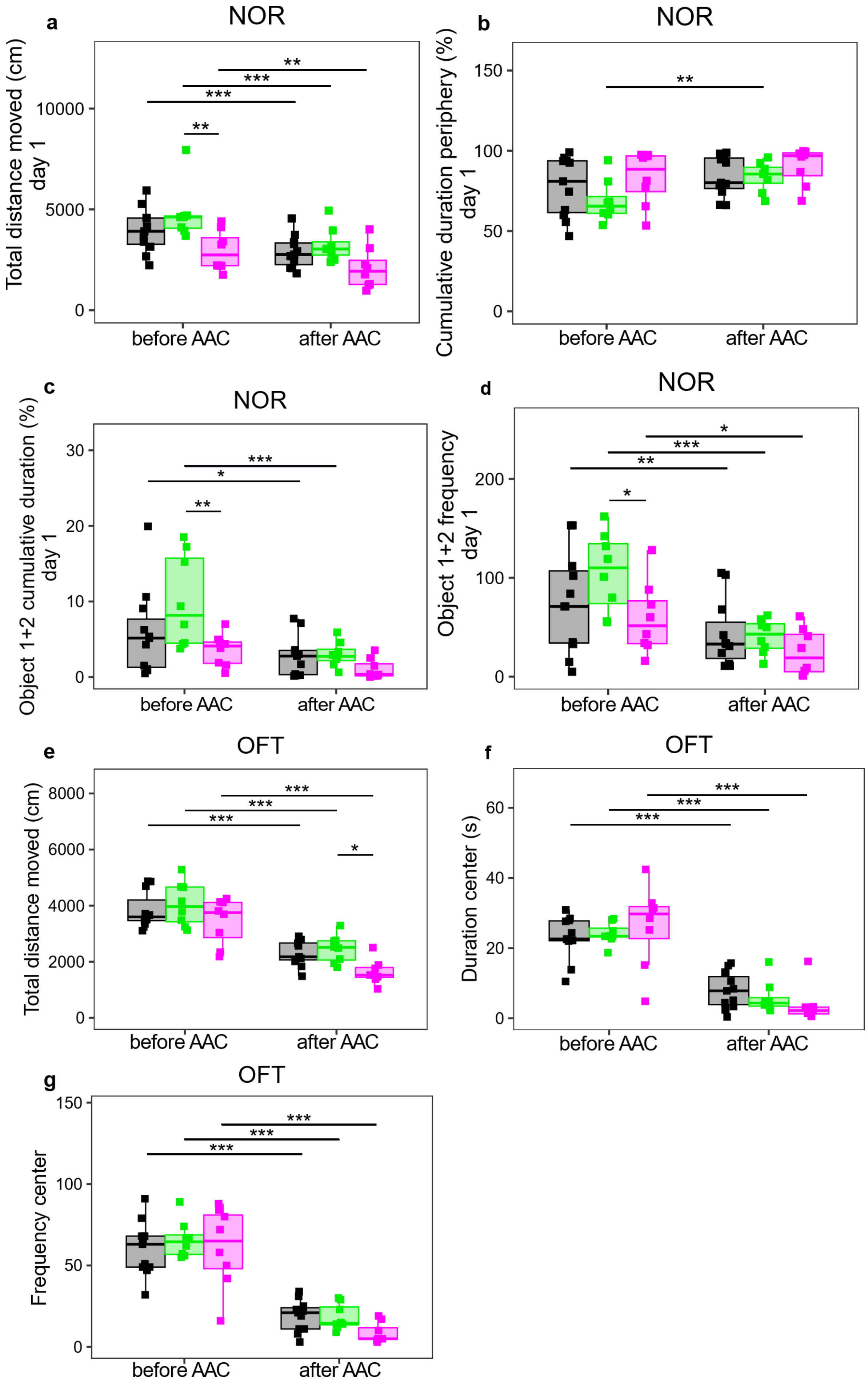
a
NOR
Total distance moved (cm)
day 1
10000
5000
0
**
***
***
**
before AAC
after AAC
b
NOR
Cumulative duration periphery (%)
day 1
150
100
50
0
**
before AAC
after AAC
c
NOR
Object 1+2 cumulative duration (%)
day 1
30
20
10
0
***
*
**
before AAC
after AAC
d
NOR
Object 1+2 frequency
day 1
200
100
0
*
***
**
*
before AAC
after AAC
e
OFT
Total distance moved (cm)
8000
6000
4000
2000
0
***
***
***
*
before AAC
after AAC
f
OFT
Duration center (s)
60
40
20
0
***
***
***
before AAC
after AAC
g
OFT
Frequency center
150
100
50
0
***
***
***
before AAC
after AAC

Reviewer Figure 3. NOR test **(a-d)** and OFT **(e-g)** were performed 1 week before and 1 week after AAC. We observed significant decrease of activity including objects approach and visits to the centre of the open field arena in sustained (magenta), phasic responders (green) and a no shock control group (black) following repeated testing. *p<0.05, ***p<0.001, linear mixed effects model followed by pairwise comparison of model means.

e. The selection of animals for the transcriptomic and electrophysiological analyses is in my view problematic, as the authors did not choose animals from each selected subgroup in a random manner. Instead, only animals with the most extreme phenotype were selected, only animals that did not shift their phenotype between MR1 and MR2, and only animals where the phenotype indeed correlated with OFT behavior. In other words, a very specific sub-set of animals was selected, which is not reflective of the whole group of phasic vs sustained responders. Based on this biased selection procedure any conclusions of the transcriptomic data for the whole groups of phasic or sustained responders is therefore not valid.

Reply: Optimally, transcriptomic analysis should be performed in the whole cohort of conditioned animals. However, this is not feasible due to practical limitations. Our rationale behind selecting animals with consistent phenotypes between MR1 and MR2 that also exhibited stable behaviour in the anxiety tests is to indeed profile and make conclusions about consistent sustained and phasic responders. We consider "shifters" as an additional phenotype. Therefore the conclusions we make from the transcriptomic analysis do not generalize to this group. We are currently planning follow-up analyses in which shifter animals will also be profiled to elucidate the biological substrate of this additional behavioural phenotype. We have provided additional details in the manuscript to clarify these points (lines 211-222, 247-248, 254-260, 448-459).

f. The high percentage of animals classified as "shifters" argues against the previous statement that the phenotype between MR1 and MR2 is highly stable and correlated. How can these two different conclusions be reconciled?

Reply: As was noted by the reviewer in the previous comment, the observed behaviour (freezing during CS) is a normally distributed continuum, mirroring the distribution of trait anxiety in the general population. While we operationalized anxiety through observable freezing behaviour, it is important to acknowledge that freezing is not a direct measure of anxiety trait, but merely a behavioural manifestation of an animal's coping strategy in a particular testing context. Therefore, trait can also be viewed as a vector of coping strategies, meaning that multiple points of assessment are necessary to make conclusions about an animal's trait. Consequently, one should anticipate the coexistence of animals exhibiting consistent coping strategies, (phasic and sustained responders), alongside those who adjust their coping strategy in case of repeated exposure to the same testing context (shifters). Nevertheless, for the majority of animals, freezing behaviour during MR2 was proportional to MR1 levels, as demonstrated by strong positive correlations observed between both time points in several independent animal cohorts (Main text Fig. 1i,j, Main text Figure 2i, Extended data Fig. 3b). It is crucial to note that further investigation into the

characteristics and implications of animals with a shifting coping strategy, although interesting, lies beyond the scope of our current study which is not a research but a resource article.

We have updated the manuscript to reflect this more accurately (lines 211-222, 448-459).

g. Transcriptomic analyses should be presented and discussed with the same statistical rigor in males vs females. It is apparent (and quite interesting), that the number of DEGs in females is a lot higher than in males. It is therefore misleading to present unadjusted p-values in the results section and figure for males, but not for females.

Reply: In the initial submission, we discussed the potential reasons underlying the differences in number of DEGs between males and females in lines 476-493. We have updated our transcriptomic analysis according to the reviewer's suggestion (lines 271-272, Main Text Fig. 3d, Extended Data Fig. 4c).

h. The low overlap of DEGs between MR1 and MR2 argues against the hypothesis that both tests are highly correlated and reflect the same behavioral state of the animals. How do the authors interpret this finding? Similarly, from the 292 overlapping DEGs, only a fraction (13) were consistently regulated. Can the authors confirm these regulations using independent samples?

Reply: Based on the time-points of tissue collection for RNA-seq (30 minutes after CS onset) we think that RNA pools reflect memory encoding and consolidation (MR1) and baseline differences (MR2) as a 30-minute window is too short to complete transcription of genes other than immediate early genes (Fowler, Sen, and Roy 2011; Phillips et al. 2023). Furthermore, a process of systems consolidation may have occurred at MR2, thereby also changing the transcriptomic profiles in the various brain areas compared to MR1. While further research is necessary to disentangle these differences, the investigation of mechanisms underlying trait anxiety was outside of scope of this resource manuscript whose main aim was to establish the behavioural model.

i. The overlap of DEGs with transcriptomic signatures of human psychiatric disorders is potentially interesting, but too little information is provided to assess this. Please include additional information on the human samples, number of input genes for each human disease, and percentage of overlap. Just referring to the two cohort papers is not sufficient. Which genes were the ones that overlapped?

Reply: In both studies, Girgenti et al. and Gandal et al. the differential expression analysis was done by using both female and male patients and controls. As we previously used DEGs from female mice only, we have now updated our analysis by using mixed DEGs from both female and male mice at MR1 for the overlap with mixed female and male disease-associated genes (Main text Figure 3i). According to the reviewer's comment, we have added a table with further study information including the number of significant DEGs (adjusted p-value < 0.05), the number of patients, and the percentage of female subjects included per disease. Additionally, we have added a table of overlapping genes between our DEGs and

the human disease-associated genes. Further information on the samples and the differential expression analysis results including log2 fold changes and p-values observed for each gene and comparison can be found in the supplementary tables and information provided by the respective studies. We have updated the manuscript according to the reviewer's comment (lines 332-338, Main Text Fig 3i, Supplementary Table 12).

Reviewer #3:

This study aimed at developing a behavioral pipeline to examine inter-individual differences in trait-anxiety in mice that could be easily adopted across laboratories. Given the higher prevalence of anxiety- or stress-related disorders in women than in men, as well as the male-dominant literature on the topic, the authors included both female and male mice in the study. The results suggested that sustained, but not phasic, fear responses during memory retrieval can be a marker of trait-anxiety both in females and males. The manuscript is well-written, and acknowledges an important, under-investigated topic about inter-individual differences in behavior. Yet, we have concerns about the behavioral profiling and some of the conclusions of the study. Specifically, whether this sort of behavioral profiling reflects trait anxiety or actually targets inter-individual differences in fear recall behavior.

Reply: We appreciate that the reviewer has very accurately outlined the relevance and value of our work.

Major comments:
Auditory aversive conditioning (AAC) findings. During the habituation day, the authors habituated the mice to the to-be-conditioned tone (10kHz, 75dB) that was later used during the auditory conditioning. They observed that female mice displayed higher freezing responses during the memory retrieval (even before CS time bins 13-24). Yet, according to the latent inhibition phenomena, pre-exposure to a neutral cue may impair its upcoming pairing with a latent stimulus. In fact, previous reports have shown sex-differences in latent inhibition, with males exhibiting more latent inhibition than females (e.g. Day et al. Neuro Lear Mem 2016, Trott et al. Lear Mem 2023, Kaplan and Lubow Psyc Res 2010). This, together with prior literature suggesting either similar or reduced Pavlovian fear memory in females compared to males (e.g., Day and Stevenson Eur J Neuro 2020), made me wonder whether it is possible that the higher fear responses ("trait anxiety") in females in this study actually relate to sex-differences in latent inhibition as a result of prior exposure to the conditioned tone? Did the authors test habituating the mice by using a different tone than the to-be-conditioned tone? Or not pre-expose to the tones at all? This would rule out any possible confounds of latent inhibition.

Reply: Exposure to the to-be-CS during adaptation day was included to measure the orienting reflex, also defensive anxiety-like behaviour manifesting as quiescence (Bradley 2009; Roelofs 2017). As we observed strong positive correlations of orienting reflex quiescence and sustained freezing during MR in both sexes (Main text Figure 1i, j) we decided to incorporate this additional test in the rest of the experiments. However, when designing the adaptation protocol, we reasoned that one exposure to the non-reinforced tone would not be sufficient for safety learning /latent inhibition (LI) to occur since it has been

previously shown that multiple exposures to the to-be-CS are necessary, with the number of exposures positively correlating to the strength of LI (De La Casa and Lubow 2001; Lipina, Rasquinha, and Roder 2011)). This principle is emphasised in the commonly used definition of latent inhibition (LI): LI is a paradigm in which a neutral cue is **repeatedly** presented in the absence of any aversive associations. Subsequent pairing of this pre-exposed cue with an aversive stimulus typically leads to reduced expression of a conditioned fear/threat response (Kaplan and Lubow 2011; D. Miller et al. 2022). Additionally, adaptation and conditioning sessions were performed in different contexts (see Methods) as it has been previously shown that LI is specific to the context in which it was administered (Miguez, Soares, and Miller 2015; R. R. Miller et al. 2015). Although, we think methodologically we took enough precaution to diminish the effects of LI to negligible if any at all, in the initial experiments conducted on male mice, we compared the effect of a single exposure to the to-be-CS before conditioning to animals without pre-exposure. Importantly, we did not detect differences in the freezing response during MR between both groups (Reviewer Figure 4a).

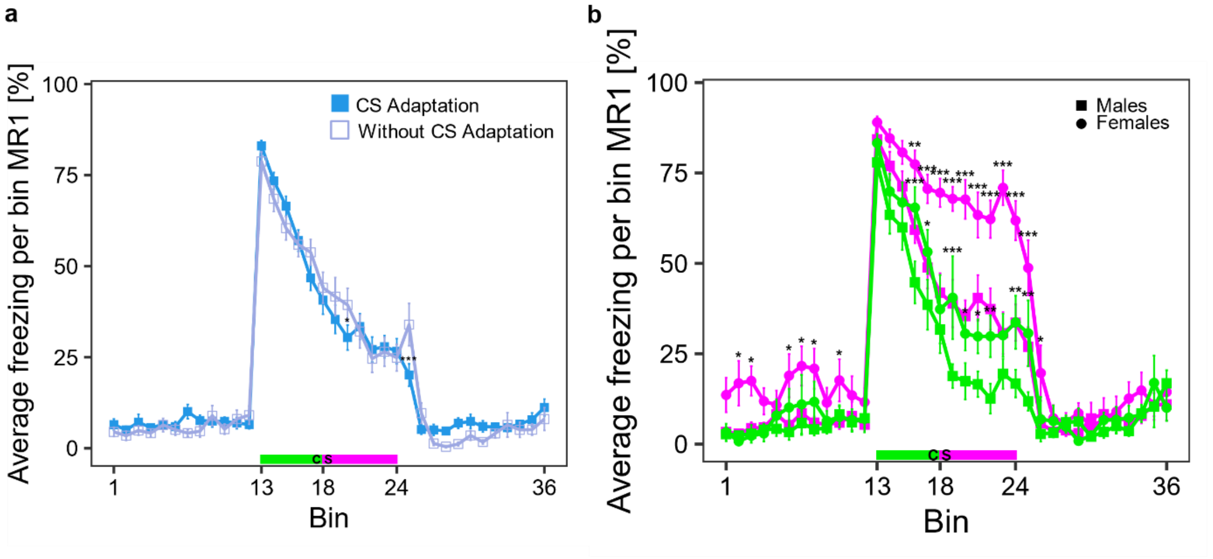

Reviewer Figure 4 a, No differences in freezing response were detected between males pre-exposed to the to-be-CS (darker blue, filled squares) and not exposed animals (lighter blue, empty squares). **b,** Sex-dependent differences in the freezing response detected in pre-CS bins 1-12 arise from increased freezing response of female sustained responders (magenta), while phasic (green) female responders did not differ from males in pre-CS freezing. * $p<0.05$, ** $p<0.01$, *** $p<0.001$, linear mixed effects model followed by pairwise comparisons of model means between animals with and without CS adaptation **(a)** or between female sustained vs. male sustained and female phasic vs. male phasic responders **(b)**.

Furthermore, we detected no significant differences in the levels of freezing during the first 90 sec of exposure during MR (Main text Figure 1f) with average freezing during bin 13 (CS-onset) reaching ceiling effect of 86% for females and 90% for males with very little inter-individual variability. Sex-dependent differences in the freezing response detected in pre-CS bins 1-12 arise from increased freezing response of female sustained responders while phasic female responders did not differ from males in pre-CS freezing (Reviewer Figure 4b).

Therefore, we think that increased freezing of females during bins 1-12 and 18-24 aligns well with higher prevalence of anxiety- or stress-related disorders in women, rather than being indicative of sex-dependant differences in LI.

Moreover we observed stronger avoidance behaviour (sustained freezing) in females not only during MRs, but also in OFT, a classical anxiety test (Extended Data Figure 1m) further confirming that detected differences in sustained freezing reflect differences in anxiety trait, not learning.

Furthermore, the authors should report the data of fear acquisition performance in the manuscript. They should compare freezing responses of sustained versus phasic responders, as well as of different sexes during fear acquisition (ideally during each training session) to determine whether groups show different memory acquisition or fear coping behavior during training already. These might predict later fear recall behavior and group allocation.

Reply: We have performed additional analysis of AAC training data. We observed significant changes of all parameters between training sessions, such as latency to first freezing event, freezing during habituation phase (contextual memory) and freezing during CS exposures indicating that the training was successful (Reviewer Figure 5). Phasic females froze less than sustained females during the habituation phase of first training session (before any CS-US pairing occurred), a difference that could potentially be attributed to differences in trait anxiety (Reviewer Figure 5c). Moreover, we detected that sustained females froze more than sustained males during the training part (CS presentation) of the first training session (Reviewer Figure 5d). However, there were no differences between these groups during the second training session, suggesting that initial differences could be attributed to different reaction to the foot shock of naïve animals (freezing vs. escape attempts). This observation is also

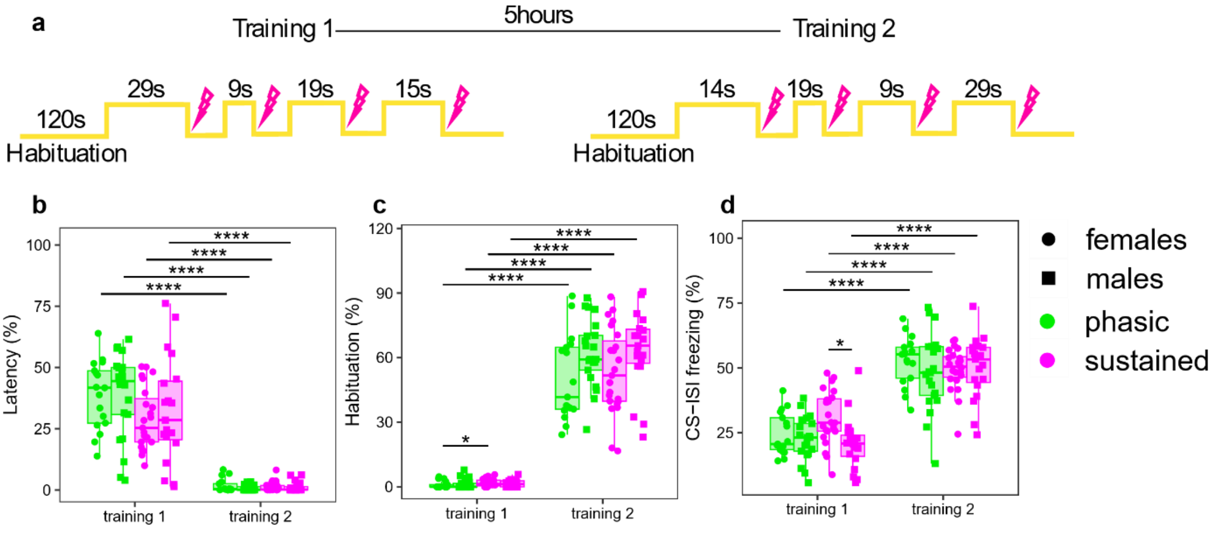

Reviewer Figure 5 No systematic sex-dependent or phenotype-specific differences were found during training. **a**, Schematic representation of the training protocol (AAC) timeline. **b-d,** No systematic sex-dependent or phenotype-specific differences in freezing behaviour were found during training for the latency to the first freezing event **(b)**, freezing during the habituation phase **(c)** and freezing during CS-ISI **(d)** (females n=40, males n=40).

consistent with females freezing more than males during MRs. Despite these observations, we did not identify any systematic phenotype or sex-dependent differences. We have updated the manuscript according to the reviewer's comment (lines 223-228, Extended Data Fig. 2a-d).

Moreover, the authors stated that different researchers performed the conditioning vs recall sessions to further distinguish them. Did the authors randomize the assignment of experimenters to conditioning or recall sessions? Did they check for an inter-experimenter effect in freezing responses? This would be particularly relevant because the authors applied different freezing thresholds for different stages of the protocol.

Reply: In the course of this project, one experimenter always performed all tests except conditioning. The second experimenter (several different people) performed the conditioning. We have now compared the effect of different experimenters that performed conditioning experiments on the sustained freezing response in female and male animals across different batches. We did not observe a significant experimenter effect in either female ($F(1,122) = 1.764$, $p = 0.187$) or male animals ($F(1,85) = 0.511$, $p = 0.477$, Reviewer Figure 6, revised manuscript lines 643-645). Within a batch, we did not randomize the assignment of experimenters to conditioning or recall for practical reasons. The rationale behind involving 2 different experimenters was to further separate the conditioning context from the retrieval context and unconditioned tasks contexts. Randomized assignment of experimenters during conditioning phase would entail the matching assignment of 2 experimenters in all other testing days.

We applied 2 different freezing thresholds: 0.5 sec for quiescence during Adaptation session and 1 sec for all other testing days (AAC, MRs). It is important to note that Adaptation and MRs were consistently performed by the same experimenter. We believe that employing different thresholds for Adaptation and MRs is justifiable. During the adaptation phase, we were measuring the unconditioned response, which tends to be more transient, manifesting as short bouts of attentive immobility that signify orienting. In contrast, conditioned freezing observed during MRs represents a behaviour more akin to "playing dead," a strategy aimed at rendering prey animals invisible to predators. Therefore, the distinction in freezing thresholds reflects the different nature of the behaviours being assessed in each phase.

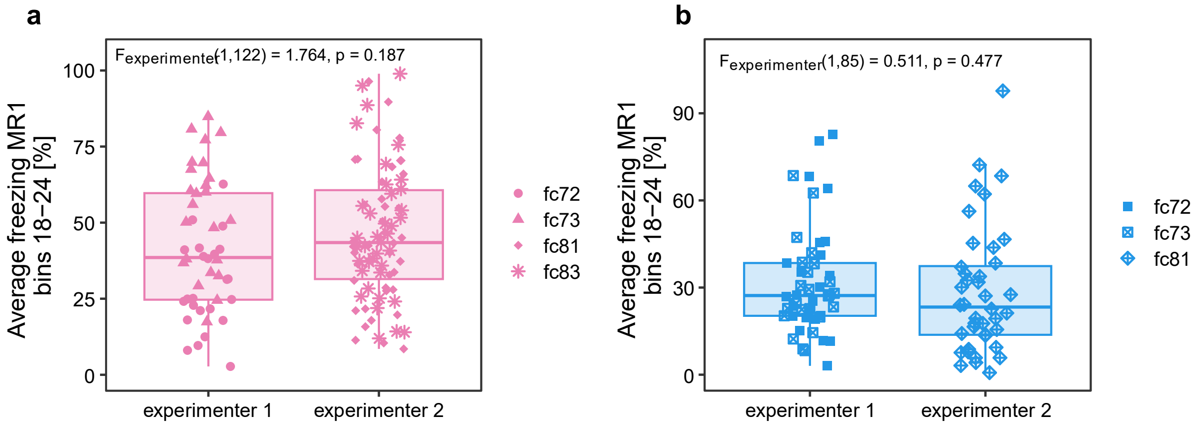

Reviewer Figure 6 No significant experimenter effect on the sustained freezing response during MR in either **(a)** female (pink) or **(b)** male (blue) mice was detected. Fc72, fc73, fc81 and fc83 represent different experimental batches. Statistical analysis was performed using ANOVA with experimenter and animal batch included as main effects. The F-statistic including degrees of freedom and the corresponding p-value for the experimenter effect is reported on each figure.

Further, the authors should give more details about how they actually defined the phasic (first 2.5 min) vs sustained response periods (2.5-6 min). Why this cut off at 2.5 min, if the tones during conditioning lasted no longer than 29 sec?

Reply: This segmentation, which refers to separating the freezing response over the 6 min of CS presentation into a phasic (first 2.5 min, bins 13-17) and sustained (2.5-6 min, bins 18-24) components, was based on two empirical observations. First, we observed that all animals reacted uniformly with high freezing in the initial CS bins however responses began to diverge during later time bins (Main text Figure 1b,c). When we quantified the variance of the freezing response during each 30s-bin, we confirmed that the variance steadily increased from bin 13 to bin 17 (0-2.5 min) and reached its peak at bin 18, remaining stable thereafter (bins 18-24) (Main text Figure 1d). Consequentially, we divided CS presentation into phasic and sustained phases. After assigning the animals to behavioural endophenotypes, we confirmed that both sustained and phasic responders reacted with similarly high freezing in the phasic part (bins 13-17), however, significant differences emerged in the sustained component (bins 18-24). Here, sustained freezers showed a significantly higher freezing response, whereas phasic freezers returned to baseline values of a no-shock control group (Extended Data Fig.2h, i).

Lastly, regarding the authors' conclusion of 'Based on the strong correlations observed in these experiments (Fig. 1i, j, Fig 2i, Extended Data Fig. 3b), we hypothesized that sustained freezing responses during prolonged retrieval sessions reflect the animal's anxiety state and can be used as a behavioral marker of an anxiety endophenotype'. It is still not clear why this method (assessing trait anxiety as a relation to the dynamics in freezing responses during retrieval sessions) is superior to other conventional anxiety-like behavior tests? Furthermore, although their conclusion is formulated as a "hypothesis", it is not fully supported by their data. The observed correlations entailed correlations between freezing responses during phasic or sustained states across multiple days, or in different time bins of MR1 and MR2, but not with other readouts of anxiety-like behavior. Yet, it is known that exposing the mice to MR1 can already influence their freezing responses in upcoming exposures (either 28d MR2, or 3d-7d MR2-MR5) because of presumed extinction of conditioned fear. Therefore, parallel to the differences in trait-anxiety, differences in extinction learning/memory can also contribute to the freezing behavior in subsequent MRs and hence may affect correlations. As such, the above-stated conclusion oversees the inter-individual differences in extinction effect. To determine how/if extinction of conditioned fear differs across sexes, the authors should also include the freezing responses in each sex in these follow-up sessions (thus not only the freezing correlation between MR1-MR5 in Fig. 1i-j).

Reply: We would like to clarify, that we are not arguing that "this method (assessing trait anxiety as a relation to the dynamics in freezing responses during retrieval sessions) is superior to other conventional anxiety-like behavior tests". The objective of this study was to design a reproducible, easy-to-adopt and translationally-relevant testing pipeline to phenotype animals according to their anxiety trait. We strongly believe that no single test can provide comprehensive information about animals' trait. Traits, defined as consistent behavioural patterns within an individual across various contexts, **necessitate evaluation through diverse tasks for effective classification**. We think the true strength of our approach lies in the combination of both conditioned and unconditioned tasks. Both conditioned and unconditioned paradigms come with their inherent limitations. Unconditioned tasks, while capturing inter-individual variability, face challenges in repeated testing with the same apparatus resulting in significantly reduced activity of animals overall (see Reviewer Figure 3), translating to limited predictive power. Conversely, performance after conditioning remains consistent across multiple retrievals, and repeated testing is standard practice. However, the low variability of conditioned responses, without modifications, renders conditioning paradigms less suitable for investigating inter-individual variability. We would like to parallel our combination approach with psychiatric assessment in humans, where the diagnostic criteria for a particular disorder are divided into sub-groups For the final diagnosis of the disorder, not all but a certain number of diagnostic points must be fulfilled. Similarly, our method provides a nuanced evaluation that does not demand adherence to all conditions.

An experiment with multiple retrievals was performed to demonstrate that sustained freezing is not randomly changing for individual animals between different retrievals as often the case for animals' performance in unconditioned tasks. In the revised manuscript, we have also included a more detailed analysis of extinction across multiple retrievals in both sexes (Reviewer Figure 7). Phasic freezing (bins 13-17) underwent significant extinction from MR1 to MR6 as indicated by the negative coefficients in a linear regression model, confirming that phasic freezing reflects a memory component (Reviewer Figure 7 a,b,e,f). At the same time, the extinction rate of the sustained component (bins 18-24) did not change significantly over time in both sexes ($b = -1.26$, $p = 0.08$ in females and $b = -1.81$, $p = 0.062$ in males) (Reviewer Figure 7 c,d,g,h), strongly indicating that while sustained freezing is also a conditioned response, its variability is driven by differences in innate trait.

Additionally, we observed that sustained freezing significantly correlated with approach-avoidance behaviour in anxiety tasks (LDT – Main Text Figure 1i,j; OFT-Main Text Figure 2g,h) and we observed multiple differences between sustained and phasic responders in anxiety tasks (Main Text Figure 2j-n). These 2 observations led us to the conclusion that sustained freezing reflects an anxiety state during MR and can be used as a marker of anxiety trait. We have updated the manuscript according to the reviewer's comment (lines 182-190, Extended Data Fig. 1g-j)

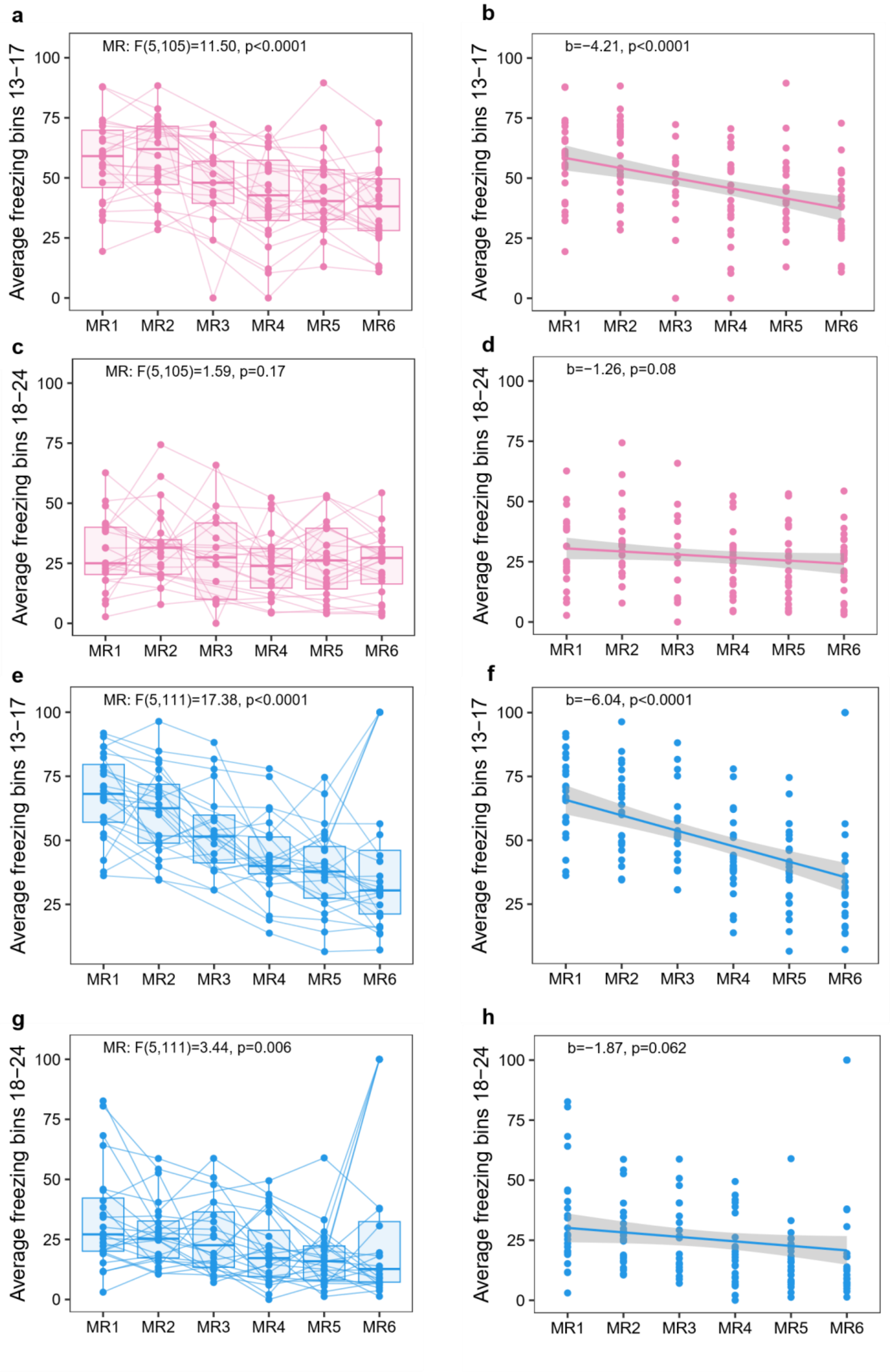

Reviewer Figure 7 Detailed analysis of extinction across multiple retrievals in both sexes. Phasic freezing (bins 13-17) underwent significant extinction from MR1 to MR6 in females **(a, b)** and males **(e, f)** as indicated by the negative coefficients in a linear regression model, confirming that phasic freezing reflects a memory component. At the same time, the extinction rate of the sustained

component (bins 18-24) did not change significantly over time in either females **(c, d)** or males **(g,h)**, confirming that while sustained freezing is also a conditioned response, its variability is driven by differences in innate trait.

Furthermore, the authors found either poor or no correlation between freezing during recall (individual bins in MR1, 2) and anxiety-like behavior in LDT (Fig 1i,j) and in EPM (Extended Data Fig. 5), respectively, prior to the conditioning. Though, they identified significant differences in EPM behavior between phasic and sustained responders (Fig 2l,n). Can the authors comment on how this is possible?

Reply: This comment further strengthens our point that single tests do not provide comprehensive information about an animal's trait. We observed batch-to-batch variability concerning correlation of performance in unconditioned tests with sustained freezing. Unconditioned tasks, more than conditioned, are influenced by the animals' internal state in the moment of testing, therefore, in our opinion, such variability is expected and normal. This is the reason why we centred our pipeline around a conditioned paradigm since it enables full control over stimulus presentation and repeated testing. Unconditioned tests initially were added to facilitate the interpretation of behaviour during the sustained phase of MR through correlational analysis. Later performance in unconditioned tests was used to select animals with consistent phenotypes for downstream analysis.

The non-significant correlation between freezing behaviour and performance in the EPM tests we observed in contrast to the significant group differences between phasic and sustained responders is explained by the additional individual variability (and potentially some random variability) in the continuous freezing measurements, which is removed when animals are assigned to a dichotomous outcome (phasic and sustained). Therefore, the correlational analysis likely lacks statistical power here. Unfortunately, for ethical and practical reasons, we are limited in the batch sizes we can include in these experiments.

Importantly, we used animals from different breeding facilities, which might have also contributed to batch-to-batch variability. Namely, in a different animal cohort, we observed strong correlations between sustained freezing and approach-avoidance behaviour in the NOR test (Reviewer Figure 1).

* Categorization of phasic or sustained responders. Several mice displayed a shift in behavior in MR2 (group shifters) compared to their categorization in MR1. This would actually argue against a trait-like feature. Is it possible to computationally predict which mice would end up in "shifter" group based on their behavior in MR1? This would ensure that the mice selected for further transcriptomic analysis only based on MR1 were actually not "shifters".

Reply: While we made sure to select animals with the most pronounced sustained or phasic phenotypes during MR1 for sequencing analysis, we cannot fully exclude that some of the animals would have been assigned to the shifter group after MR2. In a follow up project, we are indeed developing machine learning models to assign animals to different sub-phenotypes by pooling multiple batches together. However, our efforts to predict the shifter

group only using MR1 data have so far yielded models with an accuracy of 63-68%. Therefore, we do not feel confident enough to apply them yet.

Furthermore, we disagree that a shift in freezing behaviour of some animals argues against trait-like features. While we operationalized anxiety through observable freezing behaviour, it is important to acknowledge that freezing is not a direct measure of anxiety trait, but merely a manifestation of an animal's behavioural coping strategy in a particular testing context. Therefore, a trait can also be viewed as a vector of coping strategies, meaning that multiple points of assessment are necessary to make conclusions about an animal's trait. Consequently, one should anticipate the coexistence of animals exhibiting consistent coping strategies, (phasic and sustained responders), alongside those that adjust their coping strategy in case of repeated exposure to the same testing context (shifters). Nevertheless, for the majority of animals, the freezing behaviour during MR2 was proportional to MR1 levels, as indicated by a strong positive correlation observed between both time points in multiple independent cohorts (Main text Fig. 1i,j, Main text Figure 2i, Extended data Fig. 3b). It is crucial to note that further investigation into the characteristics and implications of animals with a shifting coping strategy, although interesting, lies beyond the scope of our current study, which is not a research but a resource article.

We have updated the manuscript to reflect this more accurately (lines 211-222, 448-459).

* Comparison between female responders and human post-mortem tissue: The authors increased the translational aspects of their findings by comparing their transcriptomic analysis with human post-mortem tissue analysis. However, the original paper (Girgenti et al. Nat Neuro 2021) used a mixture of women/men samples. Did the authors include the transcriptome of female/male mice to compare with the mixed human samples or used only the women data set from the human study? Furthermore, prior reports have demonstrated that the transcriptome changes upon experience. Can the authors comment on why they chose to collect tissue for transcriptome analysis 30 min after MR, but not in baseline similar to the post-mortem human tissue? Currently, the transcriptomic differences might just reflect differences in fear recall state behavior.

Reply: Indeed, in both studies, Girgenti et al. and Gandal et al, the differential expression analysis was performed by using both female and male patients and controls. As we previously used DEGs from female mice only, we have now updated our analysis by using combined DEGs from both male and female mice at MR1 for the overlap with the mixed women/men disease-associated genes (Main Text Fig. 3i). Furthermore, we have added a table showing the number of significant DEGs (adjusted p-value < 0.05), the number of patients and the percentage of female subjects included per disease. Further information on the samples and genes used for each disease can be found in the supplementary table 12 and information provided by the respective studies. We have updated the manuscript according to the reviewer's comment (lines 332-338, Main Text Fig 3i, Supplementary Table 12).

Furthermore, based on the time-points of tissue collection for RNA-seq (30 minutes after CS onset) we think that RNA pools reflect memory encoding and consolidation (MR1) and

baseline differences (MR2) as a 30 minute-window is too short to complete transcription of genes other than immediate early genes (Fowler, Sen, and Roy 2011; Phillips et al. 2023). That might also be reflected by increased correlation with a PTSD transcriptome at the MR2 time-point as this time-point is more likely to reflect a baseline transcriptome similar to post-mortem human tissue.

Ultimately, it is impossible to disentangle baseline differences in gene expression from those induced by conditioning. Furthermore, baseline brain tissue collection is unfeasible prior to AAC due to the necessity to phenotype the animals first. Nevertheless, we are arguing that differences in aversive conditioning are a direct consequence of differences in trait anxiety.

Minor comments:

Although the authors employed a series of mathematical modeling to categorize mice into phasic and sustained responses, some mice in the sustained group seem to have almost similar freezing decay, and freezing responses in the MR as the phasic fear mice. Can the authors comment on this? How can these mice (almost overlapping green and magenta data points in Fig 2g) phenotypically be so distinct? Did the authors exclude the mice in the green/magenta intersection from the further analysis?

Reply: Our clustering strategy is based on continuous measures reflecting the observation that anxiety response itself is a spectrum rather than a categorical outcome. For this reason, we performed multiple analyses on the individual level by calculating correlations between continuous measurements. Nevertheless, assigning animals to discrete subgroups has both theoretical advantages – such as increased interpretability as well as statistical advantages such as increased power due to reduced noise compared to individual measurements. Therefore, the reviewer is correct that animals in the intersection between both clusters are likely not phenotypically so distinct between each other. We did not exclude animals in the overlap between the two clusters from the behavioural analysis as we wanted to present a complete and unbiased picture and we believe that excluding these data points would have artificially augmented the differences between both phenotypes. However, for the transcriptomic and electrophysiology experiments, which were performed on a subset of animals from the complete cohorts, we carefully selected consistent phasic and sustained mice with stable phenotypes across multiple measurements as described in the manuscript (lines 247-260, Extended Data Fig. 3c-m, lines 387-389, Extended Data Fig. 5f-k).

* The authors indicated that 'Phasic responders showed an increased risk assessment behaviour in the EPM test indicated by total duration in the open arms (Fig. 2l, m) and extreme risk-taking behaviour evaluated by the significantly increased time mice spent on the open ends of the arena'. How can the authors conclude an increased or an "extreme" behavior in phasic responders if there is no "control/normal" group here? Is the comparison made towards to sustained fear group? Or did the authors check if the behavior of phasic responders statistically differed from a group of uncategorized, or mixed phasic/sustained mice? If not, this conclusion should be reformulated as it is not fully supported by their data

and could also be that "Sustained responders display reduced risk assessment and extremely low risk taking behavior compared to phasic responders".

Reply: The reviewer comment is correct. We have updated the text accordingly in the revised manuscript (lines 238-240).

* In Fig 2k the authors demonstrated that sustained responders explored the novel object less frequently. How was the total exploration duration of the novel object in the different groups?

Reply: The total exploration duration did not differ significantly in this particular batch although phasic responders showed a tendency to spend more time interacting with the object (mean = 6.12% vs. 3.43% in sustained responders, p = 0.115). As mentioned in the previous comments, we observed certain batch-to-batch variability in unconditioned tests performance. In another batch we detected differences in both total time of exploration and frequency of approach.

* The authors trained mice in the AAC training twice, each session separated by 5 h on the same day. What was the aim of second training protocol? Were the freezing responses low or insufficiently variable with single training protocol? And why did the authors select the 5 h time point? Likely that they aimed at linking the two memories by training within the temporal linkage window (<5 h), but the rationale is currently lacking in the manuscript.

Reply: We never tested a single training session protocol. The protocols were taken from Daldrap et al. (2015). It is possible that one training session would be enough, as in habituation phase of 2nd training session mice already exhibited strong freezing response to the context (Reviewer Figure 5). The 5-hour window between the 2 sessions was selected based on practical consideration to fit the training sessions in 1 day.

While we acknowledge the critical role of the conditioning protocol, we assert that it may not be pivotal in "inducing" inter-individual differences in the conditioned response. Any protocol involving **several reinforcements of the tone with mild foot shocks** could likely yield comparable results in terms of inter-individual variability during MR, particularly if memories are retrieved with prolonged CS presentation.

Furthermore, we consider that variations in the strength of the unconditioned stimulus (US) and the contingency of reinforcement (CS co-terminated with US or CS and US presented sequentially) could potentially influence the distribution of responses because these parameters might influence the subjectively determined predictive value of the CS.

* It would strengthen the validation of RNASeq results to quantify the expression of several DEGs with qPCR.

Reply: We agree with the reviewer. Unfortunately, the brain punches yielded just enough material for RNA-seq. Nevertheless, we observed a set of 292 DEGs overlapping between MR1 and MR2 which were performed in independent batches of animals with multiple

individual genes overlapping within the region which indicates replicability of our results (Main text Figure 3e).

* Given that different researchers performed different parts of the experiment, possibly the mice were priorly habituated to both experimenters. Is this indeed the case? Please add this information into the methods section as well.

Reply: In the experiments included in this manuscript mice were not specifically habituated to the experimenters as indicated in the revised manuscript (lines 642-643). In other experiments involving this pipeline, mice were habituated to handling, but we did not see any impact of that on animal behaviour. Therefore, the only protocol concerning handling of experimental animals was that in the 2 weeks preceding the experiment, mice were only handled by the 2 experimenters.

* Figure citations in the manuscript do not appear in the same order as in the figure panels. It is rather confusing to the reader.

Reply: We have made an effort to follow the order of the main figure panels in the manuscript text. However, due to the necessity to reconcile multiple factors such as the coherence in the text, as well as the logical relationship between different figure panels and last but not least, optimization of figure layout, this has not been possible in all cases for the Supplementary Figures. We hope this is acceptable to the reviewer.

* Extended Figure 1A legend should include details about the CR presentation, or what magenta/green bars represent.

Reply: We have updated the manuscript according to the reviewer's comment.

* Figure 1E should be a part of Figure 1A.

Reply: The segmentation of the freezing response into a phasic and sustained component was based on empirical observations presented in Figure 1b-d. Therefore, we showed the schematic representation of this segmentation in Figure 1e to follow the natural flow of the results.

Bradley, Margaret. 2009. "Natural Selective Attention: Orienting and Emotion." *Psychophysiology* 46(1): 1–11.

Day, Harriet L.L., and Carl W. Stevenson. 2020. "The Neurobiological Basis of Sex Differences in Learned Fear and Its Inhibition." *European Journal of Neuroscience* 52(1): 2466–86.

Fowler, Trent, Ranjan Sen, and Ananda L. Roy. 2011. "Regulation of Primary Response Genes." *Molecular Cell* 44(3): 348–60. http://dx.doi.org/10.1016/j.molcel.2011.09.014.

Honegger, Kyle, and Benjamin de Bivort. 2018. "Stochasticity, Individuality and Behavior." *Current Biology* 28(1): R8–12. http://dx.doi.org/10.1016/j.cub.2017.11.058.

Kaplan, Oren, and Robert E. Lubow. 2011. "Ignoring Irrelevant Stimuli in Latent Inhibition

and Stroop Paradigms: The Effects of Schizotypy and Gender.” *Psychiatry Research* 186(1): 40–45. http://dx.doi.org/10.1016/j.psychres.2010.07.025.

De La Casa, L. G., and R. E. Lubow. 2001. “Latent Inhibition with a Response Time Measure from a Within-Subject Design: Effects of Number of Preexposures, Masking Task, Context Change, and Delay.” *Neuropsychology* 15(2): 244–53.

Lehner, Małgorzata et al. 2010. “The Relationship between Pain Sensitivity and Conditioned Fear Response in Rats.” *Acta Neurobiologiae Experimentalis* 70(1): 56–66.

Lipina, Tatiana V., Russell Rasquinha, and John C. Roder. 2011. “Parametric and Pharmacological Modulations of Latent Inhibition in Mouse Inbred Strains.” *Pharmacology Biochemistry and Behavior* 100(2): 244–52.

Miguez, Gonzalo, Julia S. Soares, and Ralph R. Miller. 2015. “The Role of Test Context in Latent Inhibition of Conditioned Inhibition: Part of a Search for General Principles of Associative Interference.” *Learning and Behavior* 43(3): 228–42.

Miller, Dylan, Madeleine Rassaby, Katherine Collins, and Mohammed R. Milad. 2022. “Behavioral and Neural Mechanisms of Latent Inhibition.” *Learning & Memory* 29(2): 38–47.

Miller, Ralph R., Mario A. Laborda, Cody W. Polack, and Gonzalo Miguez. 2015. “Comparing the Context Specificity of Extinction and Latent Inhibition.” *Learning and Behavior* 43(4): 384–95.

Phillips, Robert A. et al. 2023. “Temporally Specific Gene Expression and Chromatin Remodeling Programs Regulate a Conserved Pdyn Enhancer.” *eLife* 12: 1–24.

Roelofs, Karin. 2017. “Freeze for Action: Neurobiological Mechanisms in Animal and Human Freezing.” *Philosophical Transactions of the Royal Society B: Biological Sciences* 372(1718).

Sartori, Simone B. et al. 2011. “Enhanced Fear Expression in a Psychopathological Mouse Model of Trait Anxiety: Pharmacological Interventions.” *PLoS ONE* 6(2).

Werka, T. 1980. “Acquisition of the Escape Reflex in Cats after the Nucleus Centralis of the Amygdala Lesions.” *Acta Neurobiologiae Experimentalis* 40(1): 433–49.
